# A High Prevalence Rate of a Positive Screen for Cognitive Impairment in Patients With Human Immunodeficiency Virus Attending an Irish Clinic

**DOI:** 10.1093/ofid/ofw242

**Published:** 2016-12-27

**Authors:** Patricia H. McNamara, Robert Coen, Janice Redmond, Colin P. Doherty, Colm Bergin

**Affiliations:** 1 Department of Neurology; 2 Department of Neuropsychology, Mercer’s Institute for Research on Ageing, and; 3 Department of Infectious Diseases, St James’s Hospital, Dublin, Ireland

**Keywords:** cognitive impairment, HAND, HIV, prevalence

## Abstract

**Background:**

Human immunodeficiency virus (HIV)-associated neurocognitive disorders occurs in 20%–50% of HIV-positive patients. We undertook this study to assess the prevalence of a positive screen for cognitive impairment in the clinic population at our institution and to demonstrate the feasibility of implementing a screening program in routine clinical encounters.

**Methods:**

This was a cross-sectional study, and patients were recruited prospectively between December 2010 and February 2013. Inclusion criteria were as follows: patients were HIV positive, over the age of 18, capable of giving informed consent, and had sufficient ability to communicate in English. Patients were screened for cognitive impairment using the Brief Neurocognitive Screen.

**Results:**

A total of 604 patients were recruited, and 51.5% had a positive screen for cognitive impairment. The majority of the study cohort were male (78.8%), mean age was 40.9 (standard deviation, 10.2) years, 70.9% were Irish, the most common mode of transmission was men who have sex with men (49.3%), 83% were on antiretroviral therapy, and 88.7% were virally suppressed. Logistic regression showed that the main factors predictive of a positive screen for cognitive impairment were the endorsement of cognitive symptoms (*P* = .024), being born in Africa (*P* < .000001), the use of benzodiazepines (*P* = .00341), being unemployed (*P* = .008), and consumption of more than 40 units of alcohol weekly (*P* = .035). There was a positive screen for depression in 9.1% and a positive screen for anxiety in 24.5%.

**Conclusions:**

The study highlights the necessity for a structured, prospective, large-scale screening program for cognitive impairment across countries with limited resources and demonstrates the feasibility of easily implementing this with minimal training.

Before the introduction of antiretroviral therapy (ART), up to 20% of patients with acquired immune deficiency syndrome (AIDS) developed human immunodeficiency virus dementia (HAD), whereas mild neurocognitive impairment was described in 30% of patients with asymptomatic human immunodeficiency virus (HIV) disease and in up to 50% of patients with AIDS-defining illnesses [1, 2]. There was a 50% decline in the rate of HAD in the post-ART era among homosexual men taking part in the Multicentre AIDS Cohort Study [3, 4]. Only 2.4% of a tertiary referral clinic HIV-positive population taking part in the CHARTER Study had HAD [5]. However, human immunodeficiency virus-associated neurocognitive disorders (HAND) continue to be an ongoing clinical issue [6, 7] despite good virological control of HIV [1].

Prevalence rates of 20%–50% of HAND have been demonstrated in large prospective studies in the United States, Europe, and Australia [5, 7–11]. Although these studies, mainly conducted in Western industrialized nations, can give us reliable data due to the comprehensive nature of evaluations, there is a need to develop screening tools that can be used during routine care because comprehensive neuropsychological testing is beyond the resources of most HIV clinics, especially in the developing world. The variation of neuropsychological batteries used and access to resources may explain the wide variation in studies in Asia and Africa, which have shown prevalence rates of 12%–60% [12–15].

Risk factors for HAND include a low nadir CD4^+^ T-cell count [5, 16], older age [16], substance abuse [17, 18], lower educational level [19], and coinfection with hepatitis C [20]. Human immunodeficiency virus dementia remains an independent predictor of time to death [21]. In a Canadian cohort study of 1651 patients observed from 1998 to 2008, HAND was associated with a higher mortality rate with a hazard ratio of 3.1 [22]. Human immunodeficiency virus-positive patients who were neuropsychologically impaired demonstrated a high rate of functional impairment in the areas of work assessment, finances, medication management, cooking, and shopping, and they were affected by a higher rate of unemployment [23].

The Brief Neurocognitive Screen (BNCS) was developed by the AIDS Clinical Trial Group to identify people with HAND. It has a reported sensitivity of 65% and a specificity of 72% in a study of 301 participants in a US study [24]. Likewise, the Hospital Anxiety and Depression Scale (HADS) has been validated for use in patients with HIV and has been used in a number of international settings [25, 26].

In this study, we used the BNCS and HADS in a cohort of diverse HIV-positive patients from a large urban center in Ireland to assess the prevalence rate of HAND in an Irish clinical setting and to show the feasibility of implementation of these screening tools in a real-world clinic. It was also envisaged that this cohort would form the basis of a more detailed study of cognitive impairment on a subgroup of patients who had a positive brief screen for cognitive impairment.

## METHODS

This was a cross-sectional observational study conducted between December 2010 and February 2013. Patients were recruited from HIV clinics at St. James’s Hospital, Dublin (SJH). Inclusion criteria were as follows: patients were HIV positive, over the age of 18, capable of giving informed consent, and had sufficient ability to communicate in English. Patients were excluded if they were intoxicated, withdrawing from alcohol or illicit substances, systemically unwell, or had other neurological disorders that prohibited testing or would explain/confound underlying cognitive impairment. Patients were screened for cognitive impairment using the BNCS, which consists of trail making tests A and B and the digit symbol test. The normative data for the Trail Making Test were taken from a meta-analysis published in The Handbook of Normative Data for Neuropsychological Assessment [27]. The normative data for the digit symbol test were taken from the Weschler Adult Intelligence Scale Revised normative data [28]. A positive screen for cognitive impairment was defined as 1 standard deviation (SD) below the population mean on 2 tests or 2 SDs below the population mean on 1 test [24]. A positive screen for anxiety or depression was a score of 11 or greater on the respective subsections of the HADS [29].

The study was undertaken during routine clinical care. Patients were treated as a presenting sample as long as they fulfilled the inclusion criteria. Each subject was invited to be part of the study during their routine visit. Testing was done by 1 researcher (P. H. M.). English competency was assessed by the researcher (P. H. M.) and based on conversation and ability to understand the information relayed, explain it back, and perform examples of each of the tests. If patients were unable to do this, they were excluded from the study. The tests took approximately 20 minutes to administer and were performed in a separate office from the routine clinic but in the same department. A power calculation was performed and yielded a minimal sample size of 302 patients, based on a total clinic population of 2200 HIV-positive patients, with a margin of error of 5%, a confidence interval (CI) of 95%, and a prevalence of HAND of 35% (chosen because it was midway between the reported international rates of 20% and 50%).

### Statistical Analysis

Statistical analysis was carried out using SPSS version 21 (SPSS Inc., Chicago, IL). Qualitative variables were presented as absolute number (N) and relative frequencies (percentages). Continuous variables were presented as absolute number, means with SDs, and medians. Descriptive analysis was carried out on qualitative variables using χ^2^ testing to examine intergroup comparisons. The Bonferroni correction method was used to adjust for multiple comparisons. Fisher’s exact test was used when cells contained less than 10 data points. Continuous variables were examined using the independent samples *t* test. When data failed to meet criteria for parametric tests, the Mann-Whitney *U* test was performed for continuous variables. All tests were 2 tailed, and statistical significance was set at *P* < .05. Multivariate analysis was performed to identify predictors of the presence of a positive screen for cognitive impairment. Binary logistic regression was carried out and the Enter method used. Only variables that were statistically significant on univariate analysis, with a *P* value < .05, were entered into the model.

## RESULTS

There were 604 patients recruited. Their characteristics are described in Table 1. There was a positive screen for cognitive impairment in 51.5%. Of note, the first 96 patients recruited to this study were also recruited to the CRANIUM study [30]. The majority of the study cohort were male (78.8%), mean age was 40.9 (SD = 10.2) years, and 70.9% were Irish. The most common mode of transmission was men who have sex with men ([MSM] 49.3%). Just over 83% were on ART, and 88.7% were virally suppressed. Over half were employed (53.5%). The median duration since diagnosis with HIV was 6.5 years, with a range of 0.03–28.9 years.

**Table 1.** Characteristics of the Study Group

Characteristics		(n = 604)	
		n	(%)
Handedness	Right	555	(91.9)
Gender	Male	476	(78.8)
Cognitive symptoms	Yes	246	(40.7)
Country of birth	Ireland	428	(70.9)
	Europe	52	(8.6)
	Africa	100	(16.6)
	Asia	8	(1.3)
	South America	12	(2.0)
	Other	4	(0.7)
English as a first language	Yes	468	(77.5)
Mode of transmission	Heterosexual	198	(32.8)
	MSM	298	(49.3)
	IVDU	93	(15.4)
	Vertical	3	(0.5)
	Other	12	(2.0)
Virally suppressed (n = 504)	Yes	447	(88.7)
Hx of hepatitis B	Yes	47	(7.8)
Hx of hepatitis C	Yes	118	(19.5)
Hepatitis C PCR positive	Yes	73	(61.9)
(n = 118)			
Hx of hypertension	Yes	67	(11.1)
Hx of hypercholesterolaemia	Yes	100	(16.6)
Hx of diabetes	Yes	15	(2.5)
Hx of syphilis	Yes	140	(23.2)
Hx of neuropathy	Yes	25	(4.1)
Hx of depression	Yes	145	(24)
Hx of anxiety	Yes	22	(3.6)
Methadone use	Yes	71	(11.8)
Benzodiazepine use	Yes	79	(13.1)
Employment history	Employed	323	(53.5)
	Student	49	(8.1)
	Unemployed	211	(34.9)
	Retired	21	(3.5)
Smoking history	Smoker	278	(46)
	Nonsmoker	214	(35.4)
	Ex-smoker	112	(18.5)
Alcohol history	None	119	(19.7)
Units per week	1–10 units	289	(47.8)
	11–20 units	100	(16.6)
	21–30 units	40	(6.6)
	31–40 units	12	(2.0)
	>40 units	21	(3.5)
	Hx of ADS	23	(3.8)
Illicit substance use	Never	385	(63.7)
	Current non-IV Use	94	(15.6)
	Current IV use	9	(1.2)
	Previous Hx	118	(19.5)
Antiretroviral therapy	Naive	95	(15.7)
	On HAART	504	(83.4)
	MTCT	3	(0.5)
	Previous exposure	2	(0.3)
Positive screen for anxiety	Yes	148	(24.5)
Positive screen for depression	Yes	55	(9.1)
Positive screen for cognitive impairment	Yes	311	(51.5)
Age (in years)	Mean (SD)	40.9 (10.2)	
	Median (range)	41 (18–77)	
Time since diagnosis (years)	Mean (SD)	8.2 (6.9)	
	Median (range)	6.5 (0.03–28.9)	
Nadir CD4 count	Mean (SD)	235.6 (102.3)	
	Median (range)	222 (1–907)	
Current CD4 count	Mean (SD)	563.3 (252.7)	
	Median (range)	541 (19–1525)	
Years of education	Mean (SD)	14.3 (3.9)	
	Median (range)	14 (0–26)	
Exposure to HAART (years)	Mean (SD)	6.1 (4.75)	
	Median (range)	4.9 (0.01–18.7)	
CPE score	Mean (SD)	7.081 (0.8711)	
	Median (range)	7 (5–13)	
HADS - Anxiety score	Mean (SD)	7.576 (4.401)	
	Median (range)	7 (0–21)	
HADS - Depression score	Mean (SD)	4.568 (4.0323)	
	Median (range)	4 (0–20)	

Abbreviations: ADS, alcohol dependence syndrome; CNS, central nervous system; CPE, CNS penetration effectiveness; HAART, highly active antiretroviral therapy; HADS, Hospital Anxiety and Depression Scale; Hx, history; IV, intervenous; IVDU, intervenous drug users; MSM, men who have sex with men; MTCT, maternal to child transmission; PCR, polymerase chain reaction; SD, standard deviation.

The results of univariate analysis are shown in Table 2. Logistic regression analysis (Table 3) showed that the main factors predictive of a positive screen for cognitive impairment were the endorsement of cognitive symptoms, being born in Africa, the use of benzodiazepines, being unemployed, and consumption of more than 40 units of alcohol weekly.

**Table 2.** Results of Univariate Statistical Analysis of a Positive Screen for Cognitive Impairment

Characteristics		Positive Screen		Negative Screen		*P* Value
		(n = 311)		(n = 293)		
		n	(%)	n	(%)	
Handedness	Right	289	(52.1)	266	(47.9)	.335
	Left	22	(44.9)	27	(55.1)	
Gender	Male	223	(46.8)	253	(53.2)	.000011
	Female	88	(68.8)	40	(31.3)	
Cognitive symptoms	Yes	139	(56.5)	107	(43.5)	.041
Country of birth	Ireland	194	(45.3)	274	(54.7)	<.000001
	Europe	21	(40.4)	31	(59.6)	
	Africa	86	(86)	14	(14)	
	Asia	3	(37.5)	5	(62.5)	
	South America	7	(58.3)	5	(41.7)	
	Other	0	(0)	4	(100)	
English as a first Language	Yes	210	(44.9)	258	(74.3)	<.000001
Mode of transmission	Heterosexual	124	(62.6)	74	(37.4)	.000012
	MSM	121	(40.6)	177	(59.4)	Std residual
	IVDU	57	(61.3)	36	(38.7)	for heterosexual
	Vertical	2	(66.7)	1	(33.3)	was 2.2
	Other	7	(58.3)	5	(41.7)	
Virally suppressed (n = 504)	Yes	221	(49.4)	226	(50.6)	.005
Hx of hepatitis B	Yes	28	(59.6)	19	(40.4)	.248
Hx of hepatitis C	Yes	73	(61.9)	45	(38.1)	.012
Hepatitis C PCR positive (n = 118)	Yes	51	(69.9)	22	(30.1)	.04
Hx of hypertension	Yes	30	(44.8)	37	(55.2)	.244
Hypercholesterolaemia	Yes	37	(37)	63	(63)	.002
Hx of diabetes	Yes	9	(60)	6	(40)	.504
Hx of syphilis	Yes	58	(41.4)	82	(58.6)	.007
Hx of cryptococcal meningitis	Yes	2	(100)	0	(0)	.169
Hx of CNS TB	Yes	5	(100)	0	(0)	.062
Hx of toxoplasmosis	Yes	7	(58.3)	5	(41.7)	.632
Hx of encephalitis	Yes	4	(80)	1	(20)	.2
Hx of PML	Yes	4	(100)	0	(0)	.124
Hx of IRIS	Yes	1	(100)	0	(0)	1
Hx of epilepsy	Yes	10	(66.7)	5	(33.3)	.299
Hx of neuropathy	Yes	6	(24)	19	(76)	.007
Hx of stroke	Yes	5	(100)	0	(0)	.062
Hx of depression	Yes	79	(54.5)	66	(45.5)	.408
Hx of bipolar disorder	Yes	4	(57.1)	3	(42.9)	1
Hx of anxiety	Yes	12	(54.5)	10	(45.5)	.77
Hx of schizophrenia	Yes	2	(100)	0	(0)	.5
Hx of psychosis	Yes	6	(85.7)	1	(14.3)	.124
Lipid-lowering therapy	Yes	22	(33.3)	44	(66.7)	.002
Antihypertensives	Yes	31	(49.2)	32	(50.8)	.702
Antiepileptics	Yes	11	(68.8)	5	(31.3)	.161
Methadone	Yes	53	(74.6)	18	(25.4)	.000032
Benzodiazepine use	Yes	65	(82.3)	14	(17.7)	<.000001
Antidepressants	Yes	42	(63.6)	24	(36.4)	.036
Antipsychotics	Yes	16	(84.2)	3	(15.8)	.004
Employment history	Employed	135	(41.8)	188	(58.2)	.000001
	Student	29	(59.2)	20	(40.8)	
	Unemployed	138	(65.4)	73	(34.6)	
	Retired	9	(42.9)	12	(57.1)	
Smoking history	Smoker	153	(55)	125	(45)	.166
	Nonsmoker	108	(50.5)	106	(49.5)	
	Ex-smoker	50	(44.6)	62	(55.4)	
Alcohol history	None	75	(63)	44	(37)	.03
Units per week	1–20 units	18.5	(47.6)	204	(52.4)	Std residual for
	21–40 units	25	(48.1)	27	(51.9)	no alcohol is 1.8
	>40 units	11	(52.4)	10	(47.6)	
	Hx of ADS	15	(65.2)	8	(34.8)	
Illicit substance use	Never	196	(50.9)	189	(49.1)	.352
	Current Non-IV Use	54	(57.4)	40	(42.6)	
	Current IV Use	5	(71.4)	2	(28.6)	
	Previous Hx	56	(47.5)	62	(52.5)	
Antiretroviral therapy	Naive	46	(48.4)	49	(51.6)	.877
	On HAART	262	(52)	242	(48)	
	MTCT	2	(66.7)	1	(33.3)	
	Previous exposure	1	(50)	1	(50)	
Positive screen for anxiety	Yes	87	(58.8)	61	(41.2)	.041
Positive screen for depression	Yes	39	(70.9)	16	(29.1)	.003
Age (in years)	Mean (SD)	39.733 (9.946)		42.126 (10.377)		.004
	Median	39		42		95% CI, −4.0173 to −0.7691
Time since diagnosis (days)	Mean (SD)	2817.675 (2302.8863)		3162.27 (2699.4141)		.338*
	Median	2365		2471		
Nadir CD4 count	Mean (SD)	221 (157.409)		251.038 (166.2)		.023
	Median	207		233		95% CI, −55.98 to −4.0915
Current CD4 count	Mean (SD)	538.116 (259.18)		590.161 (243.177)		.011
	Median	505		570.5		95% CI, −92.3022 to −11.7882
Years of education	Mean (SD)	13.865 (4.1076)		14.724 (3.66)		.012*
	Median	14		14		
Exposure to HAART (days)	Mean (SD)	2053.487 (1589.9178)		2410.078 (1861.0586)		.088*
	Median	1760		1885		
CPE score	Mean (SD)	7.069 (0.9197)		7.095 (4.1029)		.735
	Median	7		7		95% CI, −0.1791 to −0.1264

Abbreviations: ADS, alcohol dependence syndrome; CI, confidence interval; CNS, central nervous system; CPE, CNS penetration effectiveness; HAART, highly active antiretroviral therapy; Hx, history; IRIS, immune reconstitution inflammatory syndrome; IV, intravenous; MTCT, maternal to child transmission; MSM, men who have sex with men; PCR, polymerase chain reaction; PML, progressive multifocal leukoencephalopathy; SD, standard deviation; Std, standardized; TB, tuberculosis.

* Performed using Mann-Whitney *U* tests and no CIs available for the difference.

**Table 3.** Results of Logistic Regression Analysis of a Positive Screen for Cognitive Impairment

Variable	Wald	*P* Value	Odds Ratio	Confidence Interval
Symptoms	5.098	.024	1.7	1.073–2.696
African country of birth	34.051	<.000001	11.075	4.938–24.839
Use of benzodiazepines	12.828	.000341	6.746	2.373–19.176
Unemployed	6.969	.008	2.163	1.2–3.835
Alcohol consumption >40 units	4.443	.035	.228	0.058–0.902

Analysis was also carried out on the predictors of a positive screen for cognitive impairment in the patients who were all born in Ireland. All of these patients spoke English as their first language and would not have had any cultural issues with the cognitive screening tests because they would all have been accustomed to the concepts used in the screening tests through the Irish education system. In comparison to the rest of the cohort, the Irish patients were older (*P* < .001), had been diagnosed for longer (*P* < .001), had fewer years of education (*P* = .000003), had been on highly active antiretroviral therapy for longer (*P* < .001), were more likely to be male (*P* < .000001), were more likely to have acquired HIV through intravenous drug use (*P* < .000001), were more likely to be coinfected with hepatitis C (*P* < .000001), were more likely to have a history of depression (*P* = .000022), were more likely to be on methadone replacement therapy (*P* = .000003), benzodiazepines (*P* = .000006), or antidepressants (*P* = .000003), and were more likely to be smokers (*P* < .000001).

Results of univariate analysis are shown in Table 4. On multivariate analysis, the factors associated with a positive screen for cognitive impairment were intravenous drug users (IVDU) as a mode of transmission (Wald = 3.835; *P* = .05; odds ratio [OR] = 0.398; CI, 0.158–1.001), use of benzodiazepines (Wald = 11.94; *P* = .001; OR = 5.411; CI, 2.077–14.099), and being unemployed (Wald = 6.463; *P* = .011; OR = 0.398; CI, 0.158–1.001).

**Table 4.** Results of a Positive Screen for Cognitive Impairment in Irish Patients Only

Characteristics		Positive Screen		Negative Screen		*P* Value
		(n = 194)		(n = 234)		
		n	(%)	n	(%)	
Gender	Male	157	(43.3)	206	(56.7)	.041
	Female	37	(56.9)	28	(43.1)	
Mode of transmission	Heterosexual	43	(45.7)	51	(54.3)	.000486
	MSM	89	(37.7)	147	(62.3)	Std residual for IVDU was 2.7
	IVDU	55	(64.7)	30	(35.3)	
	Vertical	0	(0)	1	(100)	
	Other	7	(58.3)	5	(41.7)	
Virally suppressed (n = 504)	Yes	134	(41.9)	186	(58.1)	.00029
Hepatitis C	Yes	68	(64.2)	38	(35.8)	.000007
Hepatitis C PCR positive (n = 118)	Yes	48	(72.7)	18	(27.3)	.000003
Hypercholesterolaemia	Yes	23	(30.3)	53	(69.7)	.004
Neuropathy	Yes	4	(21.1)	15	(78.9)	.034
Stroke	Yes	4	(100)	0	(0)	.041
Depression	Yes	67	(54.5)	56	(45.5)	.016
Lipid-lowering therapy	Yes	17	(29.8)	40	(70.2)	.015
Methadone	Yes	52	(77.6)	15	(22.4)	<.000001
Benzodiazepines	Yes	62	(84.9)	11	(15.1)	<.000001
Antidepressants	Yes	39	(61.9)	24	(38.1)	.006
Antipsychotics	Yes	14	(82.4)	3	(17.6)	.002
Employment history	Employed	73	(32.6)	151	(67.4)	<.000001
	Student	7	(30.4)	16	(69.6)	
	Unemployed	105	(65.6)	55	(34.4)	
	Retired	9	(42.9)	12	(57.1)	
Smoking history	Smoker	125	(55.1)	102	(44.9)	.000037
	Nonsmoker	35	(30.2)	81	(69.8)	
	Ex-smoker	34	(40)	51	(60)	
Illicit substance use	Never	92	(38.3)	148	(61.7)	.002
	Current non-IV Use	49	(60.5)	32	(39.5)	Std residual for current non-IV
	Current IV use	5	(71.4)	2	(28.6)	use was 2
	Previous Hx	48	(48)	52	(52)	
Positive screen for anxiety	Yes	62	(53.4)	54	(46.6)	.04
Positive screen for depression	Yes	31	(67.4)	15	(32.6)	.003
Nadir CD4 count	Mean (SD)	221.375 (158.8195)		252.85 (165.0894)		.047
	Median	207.5		236		95% CI, −62.56 to −0.383
Current CD4 count	Mean (SD)	554.887 (281.9671)		603.721 (236.9321)		.007
	Median	521		587		95% CI, −98.1909 to −0.522
Years of education	Mean (SD)	13.041 (4.0679)		14.449 (3.5132)		<.001*
	Median	12		14		
HADS - Anxiety score	Mean (SD)	8.619 (4.5055)		7.333 (4.1319)		.002*
	Median	8.5		7		

Abbreviations: CI, confidence interval; HADS, Hospital Anxiety and Depression Scale; Hx, history; IV, intravenous; IVDU, intervenous drug users; MSM, men who have sex with men; SD, standard deviation; Std, standardized.

* Performed using Mann-Whitney *U* tests and no confidence intervals available for the difference.

There was a positive screen for depression in 9.1% (55 of 604) of the study group. A total of 56.4% (31 of 55) were male; 83.6% (46 of 55) were Irish; 38.2% (21 of 55) acquired HIV through heterosexual transmission; 27.3% (15 of 55) were MSM; 30.9% (17 of 55) were IVDU; 92.7% (51 of 55) were on ART; and 39 of 51 (76.5%) were virally suppressed. Median age was 40, median duration of diagnosis was 7.3 years, and median years of education was 12. There was a history of depression in 37 of 55 (67.3%) of those with a positive screen for depression, and 6 of 55 (10.9%) had a history of an anxiety disorder. Logistic regression analysis showed that a history of depression (Wald = 27.38; *P* < .001; OR = 6.717; CI, 3.291–13.71) and the use of antipsychotics (Wald = 5.119; *P* = .024; OR = 4.437; CI, 1.221–16.131) were independent predictors of a positive screen for depression. These factors likely reflect patients who had a diagnosis of depression and were on treatment for a psychiatric disorder.

There was a positive screen for anxiety in 24.5% (148 of 604) of the study group. Of the 148 patients with a positive screen for anxiety, 70.9% (105 of 148) were male; 78.4% (116 of 148) were Irish; mode of transmission was heterosexual in 33.1% (49 of 148), MSM in 45.9% (68 of 148), 19.6% (29 of 148) IVDU; 84.5% (125 of 148) were on ART; and 84.8% (106 of 125) were virally suppressed. Median age was 39, and median duration of diagnosis was 6.35 years. There was a history of an anxiety disorder in 14 of 148 (9.5%) of those with a positive screen for anxiety. There was a history of depression in 72 of 148 (48.6%) of the group with a positive screen for anxiety. Logistic regression analysis showed that female gender (Wald = 4.433; *P* < .035; OR = 1.719; CI, 1.038–2.847), a history of depression (Wald = 38.248; *P* < .001; OR = 4.655; CI, 2.859–7.578), a history of anxiety (Wald = 9.178; *P* = .002; OR = 4.68; CI, 1.724–12.703), and neuropathy (Wald, 4.747; *P* = .029; OR = 2.766; CI, 1.108–6.909) were all independent predictors of a positive screen for anxiety.

## DISCUSSION

This study was conducted in a previously unscreened population of HIV-positive subjects from a large urban center. The study population was extremely diverse. A national study was carried out looking at HIV-positive patients accessing care and from July 2009 to June 2010 in SJH, 1745 patients accessed outpatient care [31]. Our study population is fairly representative of the overall population attending SJH.

The BNCS and HADS screening tools were easy to administer and were performed efficiently as part of the routine visit with a high acceptability amongst the subjects. The prevalence rate identified in this study is in keeping with large international cohorts where more comprehensive neuropsychological evaluation was performed, and it is broadly in agreement with the validation study of the BNCS [24]. A positive screen for cognitive impairment was associated with multiple factors on univariate analysis. This highlights both the heterogeneity of the disease process and the patient group.

Within this cohort, we found that 9.1% had a positive screen for depression and that 24.5% had a positive screen for anxiety. In a European prevalence study, the rate of depression in adults was 8.56% [32]. A Canadian study examined the prevalence rate of generalized anxiety disorder in 12 312 adults over the age of 55 and found a rate of 2.8% [33].

There are a number of confounders for a positive screen on the BNCS and HADS. Substance abuse, including excess alcohol consumption, is a known risk factor for HAND [18]. Alcohol abuse and HIV have additive and interactive effects on cognition and psychiatric morbidity [34]. Excess alcohol consumption was an independent predictor of a positive screen for cognitive impairment in our HIV-positive cohort on multivariate analysis. Likewise, long-term use of benzodiazepines has been shown to affect cognition, especially visuospatial ability, speed of processing, sustained attention, and verbal learning [35]. Unemployment has been shown to be a functional consequence of HAND [5]. It was statistically significantly related to the presence of a positive screen for cognitive impairment in this study. Patients with cognitive impairment are more likely to have difficulties maintaining employment, but it is also possible that patients who are unemployed may be more likely to perform poorer on cognitive testing due to sociodemographic reasons or the coexistence of a substance abuse disorder.

A positive screen for depression and/or anxiety was also associated with multiple factors on univariate analysis. Many of the same confounders that affect the screen for HAND are also prominent here in relation to psychiatric morbidity.

Rourke et al [36] found that cognitive complaints were correlated significantly with depressive symptoms and with neuropsychological measures of attention, working memory, psychomotor skills, and learning efficiency. They found that depressive symptoms accounted for the majority of variance explained in cognitive complaints, whereas psychomotor efficiency predicted the remaining variance [36]. Bassel et al [37] studied the relationship between working memory and subjective cognitive complaints in 36 HIV-positive adults. Working memory was the strongest predictor of cognitive complaints, and depressive symptoms also independently predicted cognitive complaints [37]. This suggests that there is a complex interplay amongst cognitive impairment, depression, and anxiety, and both the dependent and independent factors that predict them.

In this study, there was a higher rate of HAND detected in those born in Africa, which may be due to language issues, lack of normative data for different countries of origin, and other cultural issues. Cultural differences and acculturation have been shown to affect the results of neuropsychological testing in African Americans compared with white Americans [38]. A recent study from Uganda showed that 68.6% of a cohort of HIV-positive patients had evidence of cognitive impairment compared with 16% of a HIV-negative control group, suggesting that there is a significant effect of HIV on cognition independent of African race and that it is not just due to a lack of culturally appropriate tests and normative data [39]. It is also possible that the higher rate of a positive screen for cognitive impairment in the patients born in Africa in this study may be due to a clade effect; however, clade status was not recorded as part of this study.

These data suggest that there is a significant public health crisis emerging in the HIV community. Worldwide infection rates are declining, although there was an increase in new HIV infections in Ireland in 2014. The fact that routine clinical encounters do not reliably detect HAND without testing suggests a potential hidden public health emergency. The lack of reliable, specific interventions for HAND, apart from encouraging compliance with ART, may suggest that little can be done; however, that assumption disregards the growing rehabilitation movement in more traditionally recognized neurodegenerative disorders, including psychosocial and educational interventions and therapies, diet and weight reduction, vascular risk modification, cognitive training, and exercise therapy. These results suggest the need for a specific intervention program for HAND with large intervention studies, including the development of pharmaceutical therapies for HAND in conjunction with ancillary rehabilitation.

The wide variety of factors that were associated with a positive screen for cognitive impairment highlights both the heterogeneity of the disease process and the patient group. It is unfortunate that none of the factors associated with a positive screen for cognitive impairment help us to further understand the pathogenesis of HAND; however, these factors highlight the complexity of cognitive impairment in HIV and the importance of cognitive screening in clinical settings.

This study has demonstrated that there was a high rate of a positive screen for cognitive impairment in a previously unscreened population attending a specialist HIV clinic in Ireland, which was associated with multiple factors on univariate analysis. The patients were a relatively asymptomatic cohort who were captured prospectively. International guidelines call for cognitive screening, but compliance is rarely or never assessed. This study highlights the feasibility of performing screening at routine clinic visits. The screening tool is easy to use and could be easily administered by clinical staff at routine clinical visits. It requires minimal training to become competent in its administration, and doctors and nurses in HIV clinics could be easily trained to administer it annually to all patients who are HIV positive.

### Limitations of the Study

There are a number of limitations of this study. First, little can be inferred about the unique effect of HIV on the brain from this study due to the lack of known specificity of the tests and the number of independent variables that may confound any test of cognitive function. Furthermore, the BNCS was not designed to draw conclusions about the severity of the impairment in those who had a positive screen. Due to its higher rate of false positives and negatives, it may be more suited to large cohort studies as opposed to individual patients [24]. Another limitation of the study is the cross-sectional nature of the study because it does not allow definitive conclusions regarding causality to be drawn and only allows conclusions regarding associations between the dependent variable and independent variables. There was no control data, unfortunately, and level of education could only be adjusted for in the trail making tests and not in the digit symbol test. In addition, there was a significant number of patients for whom English was not their first language, but it was a clinical study in a routine clinical setting. It was not possible to have adjusted normative data for the countries of birth of all the patients or to have Irish normative data on the screening tool because there is none available.

## CONCLUSIONS

This was the first large-scale, cross-sectional screening program in Ireland for cognitive impairment in the setting of HIV infection. A prevalence rate of 51.5% of a positive screen for cognitive impairment was demonstrated in this study. The study highlights the necessity for a structured prospective large-scale screening program for cognitive impairment across countries with limited resources for detailed neuropsychological analysis. It also demonstrates the feasibility of easily implementing this with minimal training. It would add approximately 15–20 minutes to 1 annual visit and ensure that patients are being screened for the potential cognitive complications of HIV. Multiple risk factors exist for HAND. Human immunodeficiency virus-associated neurocognitive disorder is likely a heterogeneous disease with differing risk factors contributing to its development in susceptible individuals. Human immunodeficiency virus-associated neurocognitive disorder and affective symptoms or disorders are likely to be interrelated and not entirely separate entities that co-occur.

Despite the complexity and interdependency of the findings and allowing for the limitations noted above, these findings are in keeping with other international studies and suggest an emerging global public health crisis with significant rates of HAND, increased survival rates, and an aging HIV-positive population. Patients with a positive screen for cognitive impairment were offered detailed neuropsychological testing to determine the presence of cognitive impairment and the neuropsychological profile.
